# Biofilms in drug-coated medical devices: Mechanisms of antimicrobial resistance and perspectives on new approaches

**DOI:** 10.1051/sicotj/2026089

**Published:** 2026-11-13

**Authors:** Mohsina Patwekar, Abhinandan Patil, Faheem Patwekar, Zulhisyam Abdul Kari, Muhammad Rajaei Ahmad Mohd Zain, Arifullah Mohammed, Sébastien Parratte

**Affiliations:** 1 Department of Agriculture Science, Faculty of Agro-Based Industry, Universiti Malaysia Kelantan 17600 Jeli Kelantan Malaysia; 2 Department of Pharmacology, College of medicine, Gulf medical university Ajman UAE; 3 D Y Patil Education Society (Deemed to be University) Kolhapur India; 4 Department of Pharmacognosy, Luqman College of Pharmacy PB 86, old Jewargi Road Gulbarga Karnataka India; 5 Advanced Livestock and Aquaculture Research Group, Faculty of Agro-Based Industry, Universiti Malaysia Kelantan, Jeli Campus Jeli 17600 Malaysia; 6 Department of Orthopaedics, School of Medical Sciences, Universiti Sains Malaysia Kubang Kerian Kelantan 16150 Malaysia; 7 Department of Biotechnology, Koneru Lakshmaiah University (KLEF) Vaddeswaram Campus Guntur Andhra Pradesh 522302 India; 8 International Knee and Joint Centre Hazza Bin Zayed St. Abu Dhabi P.O. Box 46705 United Arab Emirates

**Keywords:** Biofilm, Orthopedic implant, Prosthetic joint infection, Drug-coated device, Antimicrobial resistance, Systematic review

## Abstract

*Background*: Bacterial biofilms are a major cause of treatment failure in orthopedic implant surgery, contributing to a large proportion of chronic, device-associated infections. Biofilms contain bacteria that are able to survive antimicrobial concentrations 10–1,000-fold higher than the planktonic equivalent, and antimicrobial-coated implants, which are designed to limit this risk, can still be colonized after depletion of the antimicrobial reservoir on the implant. This systematic review aims to address how biofilm-associated antimicrobial resistance occurs on drug-coated orthopedic devices and what current and future strategies can be employed to prevent it. *Methods*: During the preparation of this manuscript, a literature base of 72 references was identified and screened for direct or translatable relevance to orthopedic implants and musculoskeletal device-associated infection, using explicit criteria. Eligible studies focused on mechanisms of biofilm formation or antimicrobial resistance or on the efficacy of device technology designed to inhibit fouling or disrupt biofilms or incorporate drugs. Non-orthopedic devices (urinary, cardiovascular, dental) were included only where they provided an example of a mechanism or coating that had a reasonable chance for translation to orthopedics and are marked as such throughout. *Results*: The formation of a biofilm on orthopedic implant surfaces occurs in five broadly accepted stages: reversible attachment, irreversible adhesion, formation of microcolonies, maturation, and dispersal, with most of the orchestration of these stages being regulated by quorum-sensing signaling. The resistance of mature biofilm is due to multiple overlapping mechanisms, including the limitation of matrix diffusion, oxygen and metabolic gradients that stimulate the growth of persister cells, upregulation of efflux pumps, enzymatic degradation of drugs, and increased horizontal gene transfer. In a pooled analysis of seven comparative studies, current drug-coated orthopedic devices (e.g., gentamicin- or silver-coated tibial nails and arthroplasty components) have been found to have a lower periprosthetic infection rate than uncoated devices (2.93, 95% CI 1.72–4.99, *p* < 0.001). Antifouling (PEG-based) surface modifications, enzymatic matrix-disrupting agents, bacteriophage-based therapies and biosensor-triggered smart implants are consistently effective in vitro and in pre-clinical models, although only limited clinical-trial evidence has been generated for most of these approaches related to orthopedic implants. *Conclusions*: The scientific basis for biofilm-related resistance mechanisms is solid and heterogeneous: the quantitative efficacy claims of next-generation coatings have been supported by in vitro or preclinical studies, but not by orthopedic clinical studies. Future clinical trials in the field would focus on novel clinical evaluation of antifouling and enzymatic methods for preventing prosthetic joint infections, standardized reporting of biofilm results, and the risk-stratified use of antimicrobial coatings to reduce unnecessary antimicrobial exposure.

## Introduction

### The biofilm–infection paradigm in orthopedic surgery

The traditional approach to infection control in orthopedic surgery has been through the use of laboratory-generated antibiotic-susceptibility data from free-floating (planktonic) bacteria. This model does not fully capture the true nature of periprosthetic joint infection (PJI) and implant-associated osteomyelitis (IAO). Periprosthetic bacteria are more commonly found in the form of biofilm attached to the surface of the metal, polyethylene, or bone cement. Biofilm-related infections represent a significant fraction of chronic and persistent infections in general, and are of particular clinical importance in immunocompromised and elderly patients and patients with implanted devices [1–3]. Antimicrobial resistance has been recognized by the WHO as a high-priority problem in global health, and biofilms are a known contributor to treatment failure and increasing healthcare expenditure [4]. Bacterial resistance phenotypes in biofilms are not only acquired resistance genes alone, but they are rather complicated physiological responses which occur as a result of several overlapping processes and susceptibility patterns that are not observed in planktonic bacteria [5].

### Drug-coated orthopedic devices: Promise and limitations

Although the orthopedic practice has been revolutionized by the use of prosthetic joints, intramedullary nails, and internal fixation devices, each of these creates one surface where biofilm may develop. To minimize this risk, drug-coated (antimicrobial-releasing) implants were developed which release antimicrobial agents at the implantation site [6]. Lower rates of device-associated infection are reported with coated compared to uncoated implants, but effect sizes are reported to be very variable depending on the type of coating technology, the patient population studied, and the study design [7, 8]. Although these improvements have been made, the formation of biofilms on coated devices after depletion of the antimicrobial reservoir or after the bacteria are able to bypass the local antimicrobial effect of the coating suggests that current approaches do not fully account for the biology of biofilm pathogenesis.

### Scope and objectives

The main aim of this systematic review is to focus on the formation of biofilm on drug-coated orthopedic implants, namely the internal fixation hardware and mainly the prosthetic joints, and on the technologies which have been suggested to prevent or disrupt the formation of biofilm on these devices, in order to examine the existing knowledge and information on the subject. Evidence from other device classes (urinary catheters, cardiovascular devices, dental implants) is only included when it has a mechanism or coating strategy that has direct translation to orthopedic practice and is pointed out as such. The aims are: (1) to summarize the mechanisms of antimicrobial resistance associated with biofilms; (2) to assess the available and emerging evidence for orthopedic device coating approaches that could limit antimicrobial resistance; (3) to identify gaps and priorities for research on orthopedic devices to achieve an infection-resistant surface.

## Methods

This review was performed and reported according to the general protocol for systematic reviews as outlined in the PRISMA 2020 statement.

### Eligibility criteria

References reported include: (a) mechanisms of the formation of bacterial biofilms or antimicrobial resistance in biofilms; (b) the performance of the drug-coated, anti-fouling or biofilm-disrupting medical device technologies, where the mechanisms have relevance to orthopedic devices or musculoskeletal device-associated infection; and (c) regulatory, clinical or implementation aspects related to antimicrobial-coated orthopedic devices. References that dealt with a purely environmental or non-clinical context, or a clinical context and device type that did not have a plausible relationship to orthopedics were not included.

### Information sources and search strategy

The literature was gathered by targeted searches of PubMed/MEDLINE, Scopus, and Google Scholar, using combinations of the following terms: “biofilm”, “orthopedic implant” OR “prosthetic joint infection” OR “periprosthetic joint infection”, “drug-coated” OR “antimicrobial coating” OR “antifouling”, and “antimicrobial resistance” OR “antibiotic tolerance”, between 2002 and January 2026. Hand searching of retrieved review reference lists was performed to find further eligible reviews.

## Results

### Biofilm structure and composition

The bacterial biofilms are three-dimensional, and cells communicate via cell-to-cell signaling within them and have programmed gene expression. The extracellular polymeric substance (EPS) matrix, which can consist of polysaccharides, proteins, extracellular DNA (eDNA), and lipids, usually makes up a significant proportion of the biomass in a biofilm, with exopolysaccharides being a large proportion of the matrix weight; composition is different for various species and environments [9–11]. Matrix polysaccharides are not merely structural: their largely anionic charge creates a polyelectrolyte environment that influences diffusion of charged antimicrobial molecules, including several agents used in orthopedic antimicrobial coatings [12]. eDNA additionally functions as a reservoir for horizontal gene transfer, facilitating the spread of resistance determinants within biofilm populations on implant surfaces [13].

### Developmental stages and temporal dynamics

It has been widely suggested that the growth of a biofilm on an implant surface follows a five-step process (Figure 1). Reversible attachment (approx. 0–2 h) is accomplished by weak Van der Waals and hydrophobic interactions and may be disrupted at this stage [14, 15]. Initial EPS deposition and upregulation of adhesive structures like pili and fimbriae are followed by irreversible adhesion (approx. 2–6 h) [16, 17]. Active bacterial division and appearance of quorum-sensing–coordinated gene expression occur during early micro-colony formation (approx. 6–24 h). The characteristic “tower and channel” structure found in mature biofilm can be seen after late maturation (approx. 24–72 h) [18, 19]. After 72 h, a dispersal phase will produce motile daughter cells that are able to colonize new surfaces, while the remaining biofilm will contain metabolically dormant persister cells, which are a known source of recurrent periprosthetic infection [20, 21].

**Figure 1:**
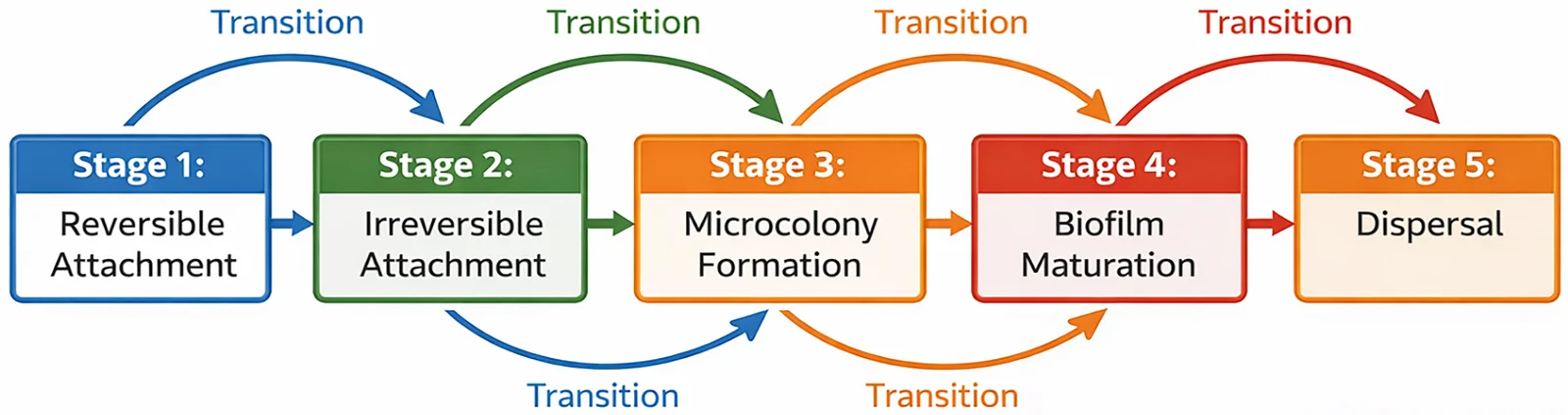
Biofilm development in five stages. The development of bacterial biofilms is stereotyped by a 72+ hour’s developmental program. Stage 1 (0–2 h): surface binding due to van der Waals forces and hydrophobic interactions, which is reversible. Stage 1 (0–2 h): Surface binding is reversible, via van der Waals forces and hydrophobic interaction. Stage 2 (2–6 h): is irreversible, includes up-regulation of adhesins and fimbriae. Stage 3 (6–24 h): is the stage of the development of microcolonies, as bacteria divide actively and exopolysaccharides are produced in great amounts. Stage 4 (24–72 h): complete biofilm organism – tower and mushroom shape; water channels are formed to distribute nutrients. Stage 5 (>72 h): dispersal stage, produces motile and chemotactically active cells, stores metabolically dormant persister cells.

### Quorum sensing and coordinated gene regulation

Biofilm formation, expression of virulence factors, and resistance phenotypes are regulated by quorum sensing, the density-dependent diffusible autoinducer molecules [22, 23]. In the orthopedic device infection pathogen, Pseudomonas aeruginosa, two relevant hierarchical signaling systems, Las and Rhl, control overlapping but different gene sets, some of which are directly associated with mechanisms of antimicrobial resistance (Figure 2) [24, 25].

**Figure 2:**
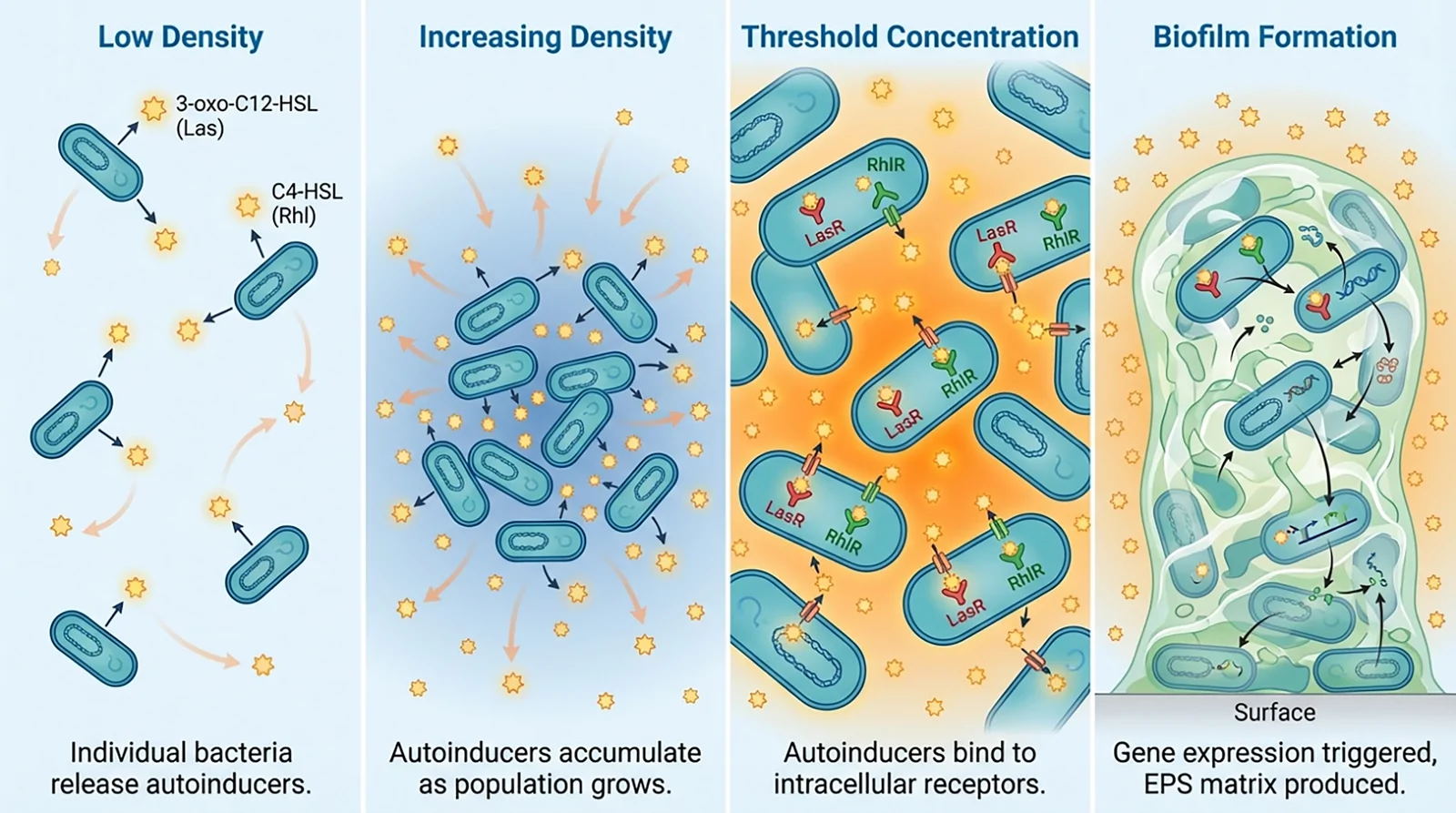
Quorum sensing mechanism. Bacterial communication through density-dependent signals leads to coordinated multicellular behavior in bacterial biofilms. Panel 1: Single bacteria generate small autoinducer molecules (N-3-oxododecanoyl-L-homoserine lactone when Las system is involved; N-butyryl-L-homoserine lactone when Rhl system is involved) which are small in size and diffusive. Autoinducers: Panel 2: There is an increase in accumulation of autoinducers with cell density in the biofilm microenvironment. If the autoinducers are allowed to reach a threshold concentration, then the cognate regulatory proteins (LasR, RhlR) will bind to them and will lead to a signal transduction cascade. Panel 4: Coordinated expression of biofilm-specific genes including virulence factors, EPS production, and, most importantly, genes encoding antimicrobial resistance determinants, which provide a direct molecular link between biofilm formation and the resistance phenotype.

### Mechanisms of biofilm-mediated antimicrobial resistance

The resistance mechanisms that occur in mature biofilms are not mutually exclusive – they all contribute to the overall resistance, either acting together or as independent processes (Figure 3): (i) certain antimicrobials are excluded from the biofilm by the EPS matrix because of their size, charge, and hydrophobicity [25–27]; (ii) the presence of oxygen and nutrient gradients within the biofilm creates metabolically quiescent subpopulations that are poorly susceptible to antibiotics that require active metabolism [28–30]; (iii) the environment of the biofilm favors the development of phenotypically tolerant, but not genetically resistant, persister cells that can repopulate the biofilm after antibiotic exposure [31–33]; (iv) enzymes produced by the biofilm can locally degrade antibiotics; (v) expression of efflux pumps is often upregulated within the biofilm; and (vi) the environment of the biofilm favors horizontal gene transfer, which increases the spread of resistance genes within the implant-associated bacterial population [34, 35].

**Figure 3:**
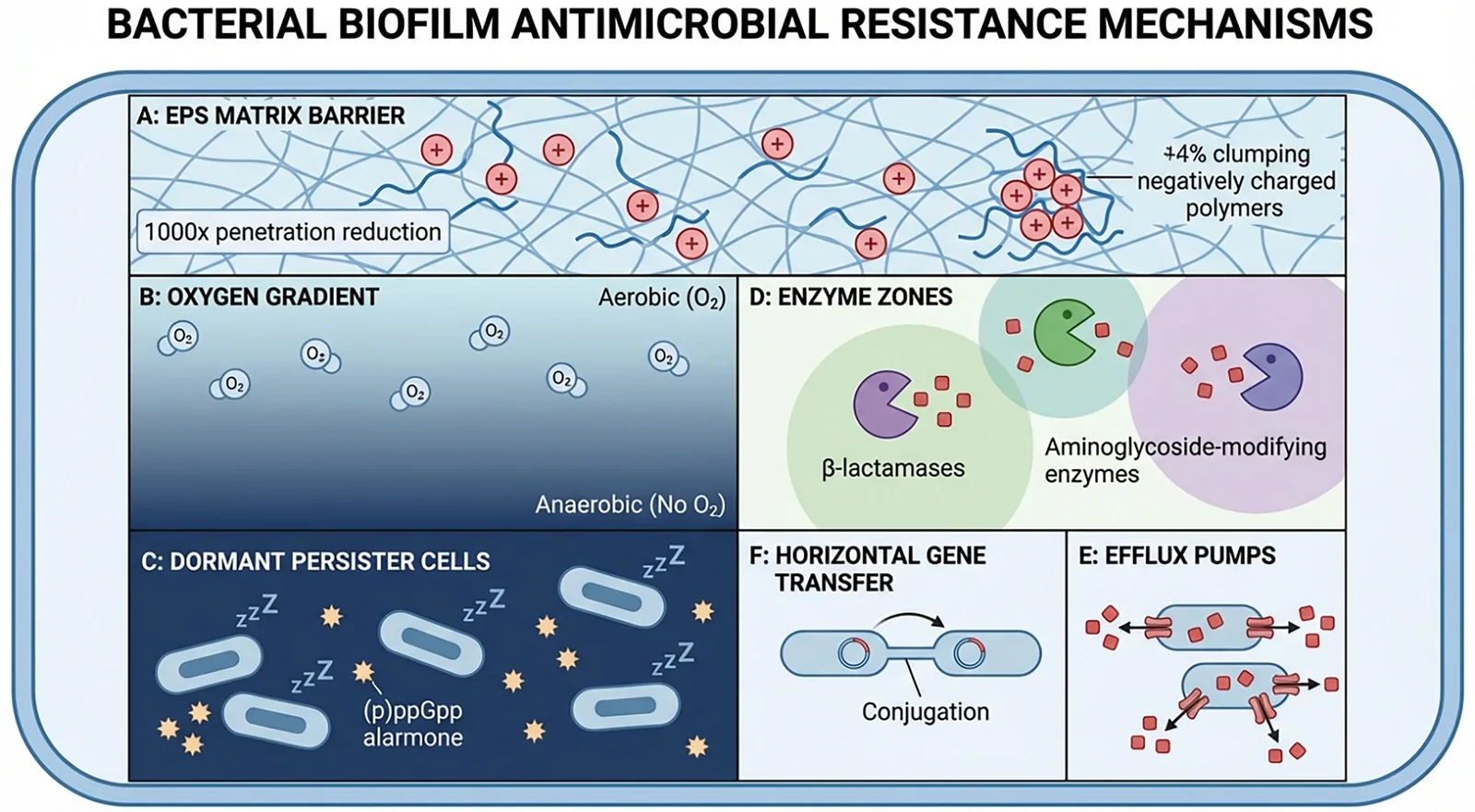
Antimicrobial resistance through biofilm. Based on a 10–1,000-fold increase in resistance, this is achieved by a combination of six synergistic complementary mechanisms. Panel A: Extracellular polysaccharide matrix forms a physical barrier, which is only partially permeable to charge and hydrophobicity; negatively charged polymers bind cationic antibiotics (aminoglycosides, polymyxins) and inhibit penetration by about 1,000 times. Panel B: Oxygen concentration gradients developed in biofilm having aerobic outer and hypoxia-anaerobic inner layers. In anaerobic environments, microorganisms produce the alarmone (p)ppGpp, leading to the production of metabolically dormant persister cells that are resistant to antibiotics that require metabolism. Panel D: Biofilm-associated enzymes (β-lactamases, aminoglycoside-modifying enzymes) form degradation areas, destroying penetrating antibiotics. Biofilms upregulate expression of the efflux pumps (RND family, ABC transporters) which expel intracellular antibiotics. Resistance determinants are spread rapidly by horizontal gene transfer through the process of conjugation (Panel F) and by phage-mediated transduction (Panel G) (10–1,000-fold higher rates).

### Current drug-coated orthopedic devices: Approaches and limitations

Antimicrobial orthopedic devices include surface adsorption or polymer-matrix encased drugs [36–40]. Gentamicin or other aminoglycosides can be applied as surface-adsorbed agents, where high initial local concentrations are present, but quickly wash off with the onset of early bacterial colonization risk, and thus may increase the risk of subtherapeutic exposure that could lead to resistance [41–43]. More sustained release can be achieved using polymer-matrix encapsulation (e.g., PLGA, PLA, PCL carriers), which has been used in orthopedic implants and drug-eluting devices in general (Figure 4) [44–46]. Despite the fact that there is a relatively small amount of clinical trial evidence for orthopedic devices compared to the number of preclinical studies, the findings in this small number of comparative studies were consistent, showing that antimicrobial-coated orthopedic implants were associated with significantly lower periprosthetic infection rates than uncoated implants in the seven studies included (OR 2.93, 95% CI 1.72–4.99, *p* < 0.001) [7, 8, 40].

**Figure 4:**
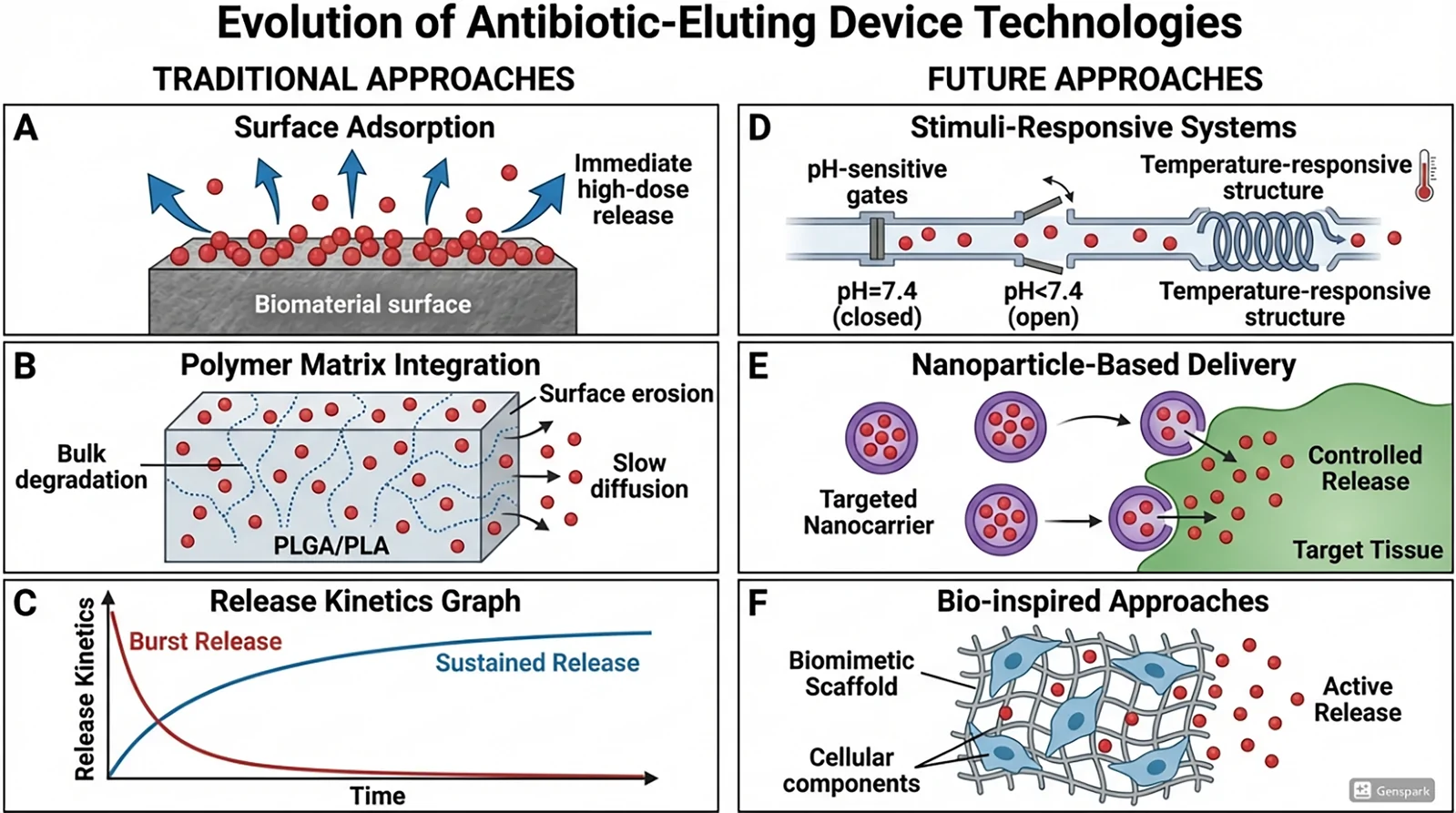
Left panels depict a traditional antibiotic-eluting device. Panel A: Surface adsorption approach with direct antibiotic coating with rapid elution (minocycline/rifampin systems); the mechanism generates high concentrations at the beginning but is rapidly depleted. Panel B: Surface erosion and bulk degradation of individual particles of polymer matrix (PLGA, PLA, PCL) that encapsulate antibiotics and exhibit sustained release over weeks. Panel C: Kinetics of release: burst release curve (surface adsorption) and sustained release profile (polymer matrix). The future approaches are shown in the right panels. Panel D: Polymers that are stimuli-responsive, releasing drugs in response to changes in pH (inflammatory microenvironment) or temperature. Panel E: Enzyme-immobilized surfaces and coatings that enable the enzyme (alginate lyase enzyme or proteases) to be bound to the surface to enable the degradation of the biofilm matrix and penetration of the antimicrobial. Panel F: Intelligent implants: Contain inbuilt biosensors, which can sense the quorum sensing molecules and only be able to release antimicrobials when the formation of biofilm begins.

### Antifouling and enzymatic coating strategies

Antifouling surface modifications that do not release antimicrobial agents (e.g., PEG-based coatings) are the least studied type of antifouling modification and have the advantage of not causing antimicrobial resistance. This approach is supported mechanistically but not by direct orthopedic outcome data because there are no orthopedic-specific antifouling trials, and the evidence for non-orthopedic devices like urinary catheters suggests the approach may yield reductions in catheter-associated urinary tract infection (CAUTI) rates in comparative studies [47–52]. Silver nanoparticle coatings are shown to be antimicrobial, with size-dependent activity, and have been tested in an orthopedic implant setting, but the potential for silver toxicity and resistance has tempered enthusiasm for widespread clinical use [53, 54]. Enzymatic biofilm disintegration using enzymes like alginate lyase to disrupt the matrix has yielded significant reductions in biofilm biomass in vitro, but the magnitude of reduction has only been reported for in vitro biofilm models, and the stability and immunogenicity still need to be resolved at the transition stage [55–58].

### Emerging technologies

Bacteriophage-based therapy involves the use of depolymerase enzymes encoded in the bacteriophage genome that disrupt the biofilm matrix, enhancing penetration of biofilm by antibiotics and killing of the bacteria while preserving commensal bacteria; although there are some pre-clinical studies in orthopedics, there has been minimal translation to clinical use [59, 60]. Early-stage, but mechanistically promising, approaches that involve the use of biosensor-based smart implants capable of detecting the onset of biofilm formation and then releasing antimicrobial agents at specific sites, and combination therapies involving the use of enzymes that degrade EPS or quorum-sensing inhibitors in combination with traditional antibiotics are specifically mentioned in the orthopedic device literature (Figure 5) [61–64].

**Figure 5:**
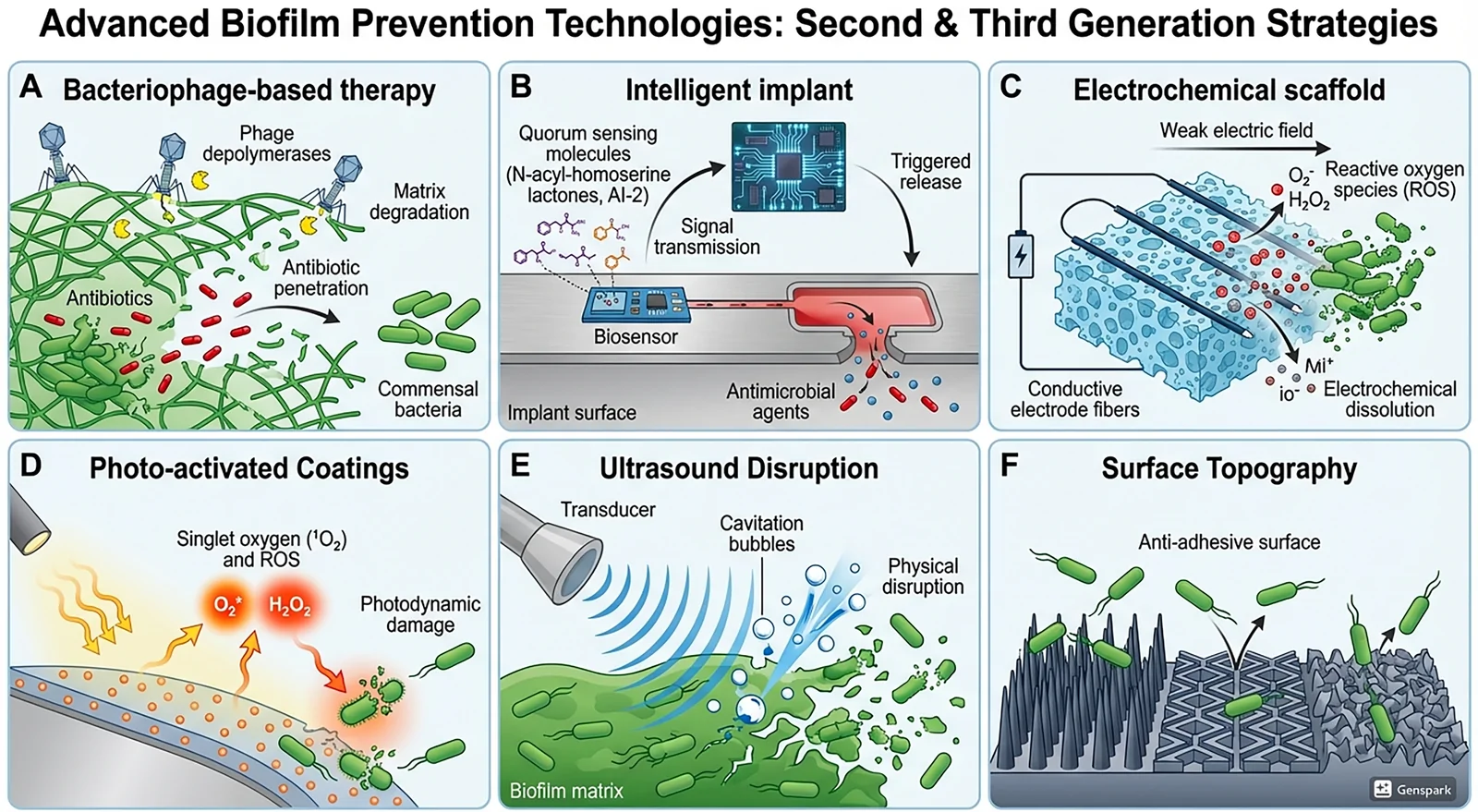
New Advanced Biofilm Prevention technologies. Second-generation strategies combine a number of complementary mechanisms. Panel A: Bacteriophage-based therapy using depolymerases produced by the bacteriophage to break down the polysaccharides in the biofilm matrix, allowing antibiotics to penetrate mature biofilms; specificity of the bacteriophage prevents destruction of commensal microbiota. Panel B: Intelligent implants containing biofilm sensing, locally embedded biosensors which release increased amounts of antimicrobial agents on demand when transmitting the presence of active biofilm growth (bacterial quorum sensing molecules: N-acyl-homoserine lactones, autoinducer-2). The first panel has stimuli-responsive polymers which can provide temporal-spatial control: Panel C: pH-responsive release in the acid inflammatory microenvironment, temperature-responsive poly (N-isopropylacrylamide) triggers a phase transition in body temperature, light-responsive coatings provide external control. Panel D: Enzyme-immobilized surfaces with enzyme immobilized on surface which has alginate lyase from Pseudomonas and protease to matrix proteins which inhibited the formation of biofilms by 80–90%. Panel E: Coatings that release immunomodulatory, toll-like receptor (TLR) agonist, interferon-g (IFN-g) or chemokine. Panel F: Antifouling Surface: Multifunctional with activity to disrupt the matrix with enzymes, antimicrobial and immune enhancing.

### Regulatory, clinical, and implementation considerations

Biocompatibility testing, durability testing to ensure long-term antimicrobial efficacy, and post-marketing surveillance are required for regulatory pathways of orthopedic devices that have an antimicrobial coating [65]. The in vivo performance of biofilm prevention strategies has always been lower than in vitro performance and has been influenced by the host immune response, intraoperative contamination, and polymicrobial infection [66]. A known risk of unnecessary antimicrobial exposure exists for using antimicrobial-coated devices in low-risk procedures, and potential downstream effects of the development of antimicrobial resistance have been proposed, with risk stratification of use advocated to balance infection prevention with the risk of unnecessary antimicrobial exposure [67, 68].

## Discussion

The in vivo and in vitro evidence for matrix-mediated diffusion limitation, metabolic gradients that promote the development of persister cells, enzymatic drug inactivation, upregulation of efflux pumps, and horizontal transfer all contribute, to varying extents and usually in combination, to the large resistance phenotype of bacteria growing within biofilm. There is less consistency in evidence for next-generation prevention strategies. Antifouling and enzymatic methods seem to be effective in vitro, but have little direct orthopedic clinical evidence other than the results from urinary catheter trials. Orthopedic applications of smart, biosensor-activated implants, as well as phage-based therapeutics, are still at a very early stage. This “efficacy to in vivo clinical benefit” translational gap, which is common throughout the wider biomaterials-infection literature [64, 65, 67] was identified as the main challenge across the literature included here. There was less discussion of personalized approaches, such as patient-specific infection-risk profiling or immunomodulatory coatings to the host; these techniques were discussed in a lesser number of included studies but still represent a nascent but promising direction rather than a proven one.

### Limitations

There are a number of limitations to this review. A methodological limitation compared to a systematic review following the preferred procedure of two independent reviewers with third-party adjudication of differences is that the author team performed screening and eligibility assessment, rather than the latter. A formal risk-of-bias and/or quality assessment tool (e.g. ROBINS-I for non-randomized studies, GRADE for certainty of evidence) was not used, due to the heterogeneity of study designs included (in vitro, non-randomized and clinical); effect estimates should be considered with this. Although a formal meta-analysis of the whole evidence base was not undertaken due to the diversity of outcome measures used across the included studies, a previously published meta-analysis is cited directly where applicable (Section “Current drug-coated orthopedic devices: Approaches and limitations”). The search was only for English-language, peer-reviewed publications, and may not have included relevant non-English evidence.

## Future perspectives and research priorities

Recommendations from the literature included in this report are: future randomized, adequately powered, clinical trials of new antifouling/enzymatic coating strategies in prosthetic joint and internal fixation populations; the need for standardized outcome reporting in orthopedic device biofilm studies for future meta-analysis; the need for continued development of the theory of persister cell biology and horizontal gene transfer in orthopedic device biofilms; and the need for risk-stratified deployment of antimicrobial coatings, reserving intensive antimicrobial strategies for higher risk patients (to avoid unnecessary antimicrobial exposure). The most frequently suggested methods for improving clinical outcomes across the literature reviewed included combinatorial approaches such as pairing antifouling chemistry, enzymatic disruption of matrices, and immune-modulating coatings [68].

## Conclusions

The main reason for treatment failure on drug-coated orthopedic implants is still due to biofilm formation. Several mechanisms are responsible for the resistance of bacteria within biofilms, and existing antimicrobial-coated devices, which have been shown to reduce infection rates compared with uncoated devices, are not able to eliminate this risk. While each of these approaches has potential mechanistic benefits, there is limited clinical evidence for most of these approaches specifically in orthopedics. The proposed orthopedics-specific clinical evidence is limited for most of these approaches, but each has plausible mechanistic advantages. Advancement of infection-resistant orthopedic devices will rely on future clinical testing of these technologies, consistent reporting of outcomes, and ongoing cooperation between microbiologists, biomaterials engineers, orthopedic surgeons, and regulatory scientists.

## Data Availability

Not applicable – this is a literature-based systematic review; the full list of screened and included/excluded records is available from the corresponding author on request.
